# Pulmonary barotrauma in SCUBA diving-related fatalities: a histological and histomorphometric analysis

**DOI:** 10.1007/s12024-022-00567-1

**Published:** 2023-01-27

**Authors:** Josep M. Casadesús, Javier Nieto-Moragas, Maria T. Serrando, Pere Boadas-Vaello, Ana Carrera, Fernando Aguirre, R. Shane Tubbs, Francisco Reina

**Affiliations:** 1 Institute of Legal Medicine and Forensic Sciences of Catalonia (Division of Girona, Spain), Av. Ramón Folch, 4-6, Girona, 17001 Spain; 2 Research Group On Clinical Anatomy, Embryology and Neuroscience (NEOMA), Department of Medical Sciences, University of Girona, Girona, Spain; 3 Experimental Neurophysiology and Clinical Anatomy (NE&AC; 2017 SGR 01279), Department of Medical Sciences, University of Girona, Girona, Spain; 4 Department of Medical Sciences, Faculty of Medicine, University of Girona (Spain), Av. Emili Grahit 77, Girona, 17003 Spain; 5 ICS-IAS Girona Clinical Laboratory, Av. Dr. Castany S/N, Salt, 17190 Spain; 6 Special Group for Underwater Activities (GEAS), Spanish Civil Guard, C/Torroella S/N, Estartit, 17258 Spain; 7 Department of Neurosurgery, Tulane Center for Clinical Neurosciences, Tulane University School of Medicine, New Orleans, LA USA; 8 Department of Neurology, Tulane Center for Clinical Neurosciences, Tulane University School of Medicine, New Orleans, LA USA; 9 Department of Structural & Cellular Biology, Tulane University School of Medicine, New Orleans, LA USA; 10 Department of Neurosurgery and Ochsner Neuroscience Institute, Ochsner Health System, New Orleans, LA USA; 11 Department of Anatomical Sciences, St. George’s University, St. George’s, Grenada

**Keywords:** SCUBA diving, Drowning, Pulmonary barotrauma, Arterial gas embolism, Morphometric histology

## Abstract

Arterial gas embolism following pulmonary barotrauma occurs in 13–24% of cases of diving deaths. The study aimed to evaluate the usefulness of a histomorphometric digital analysis in the detection of air space over-distension due to pulmonary barotrauma. The study was performed on lung parenchyma specimens of 12 divers: six had died due to arterial gas embolism following pulmonary barotrauma (mean age at death of 54 years, range of 41–61 years), and six had drowned in saltwater without a diagnosis of pulmonary barotrauma (mean age at death of 54 years, range of 41–66 years) (positive controls). For negative controls, six cases of non-SCUBA divers (mean age of death of 42 years, range of 23–55 years) who died of intracerebral haemorrhage were evaluated. No significant differences were observed in the characteristics of the air spaces between control groups (positive and negative). However, differences were observed in the area occupied by air spaces and the percentage of air space area when we compared the case group to the controls (*p* < 0.01); and there was a slight difference in the maximum and minimum diameters of air space (*p* < 0.05). The mean area occupied by air spaces and the mean percentage of air space were the most useful for discriminating pulmonary barotrauma from other causes of death (100% sensitivity and 91.7% specificity). Based on our study, inclusion of an increased pattern of air spaces as a possible diagnostic criterion for pulmonary barotrauma would be useful in discerning the cause of diving death.

## Introduction

Self-contained underwater breathing apparatus (SCUBA) diving is one of the most popular and practised underwater activities [1, 2], and although it is overall a safe activity [3, 4], diving accidents can be potentially serious and even fatal [1, 5]. Decompression illness (DCI) is caused by intravascular or extravascular bubbles that are formed as a result of a reduction in environmental pressure [6]. The term covers both decompression sickness (DCS) and arterial gas embolism (AGE) [7], and while DCS is caused by evolution of bubbles from dissolved inert gas, AGE is caused by introduction of air into the systemic circulation by pulmonary barotrauma (PBt) [8–10]. Currently, different terms have been used to refer to a PBt. These include pulmonary overexpansion injury, pulmonary over-inflation syndrome, extra-alveolar air syndrome and intrathoracic hypopressive syndrome [9, 11–13]. However, each of these diagnoses has different clinical manifestations such as subcutaneous emphysema, pneumomediastinum, pneumothorax, pneumoperitoneum or AGE, including the death of the diver within minutes [8, 9, 14–16].

Although drowning is the main cause of death, arterial gas embolism following pulmonary barotrauma (PBt/AGE) during exposure to reduced environmental pressure (PBt of ascent) has been described in 13–24% of cases [17, 18], and this is well documented in diving medical texts [2, 9, 19–21]. According to Boyle’s law, when a SCUBA diver ascends to the surface too quickly without exhaling appropriately, the gas retained in their lungs increases the intrapulmonary pressure while external pressure decreases rapidly [12, 14, 15, 22, 23]. In an emergency situation, panic worsens the diver’s breathing and sometimes can close the glottis completely. Laryngospasm, bronchoconstriction (if water is inspired) or asthmatic bronchospasm has also been described as potential causes that might prevent air exhalation [12]. In addition, other findings such as mucous plugs or foreign bodies may make airflow difficult, even expert divers. This breathing gas remains inside the lungs at a pressure higher than the environmental pressure, converting the thoracic cavity into a pressure container [14]. This increase in intrapulmonary lung tension results in a pulmonary histopathological pattern characterized by over-distension of the air spaces (AS) distal to the terminal bronchiole and this leads to bronchoalveolar emphysema with rupture of the alveolar air sacs. Therefore, gas is then introduced into the blood vessels which are no longer intact [8, 9, 13, 24]. Currently, it is agreed that alveolar tears could provide the avenue for entrance of air into the stroma of the lung. This air develops an interstitial emphysema within a perivascular sheath [8, 13, 25] defined as cystic spaces inside the lung interstitial tissues [26], and allows air to escape into the pulmonary venous system, thereby accessing the left side of the heart [8, 13, 17, 18]. If air bubbles reach the cerebral arterial circulation, they can interrupt blood flow to the brain, and cause AGE with ischemic injuries such as brain anoxia or even death [27].

In order to establish a PBt/AGE post-mortem diagnosis, it is essential to know the deceased person’s dive profile, to use specific autopsy techniques and/or image diagnoses, to obtain various toxicological and histopathological studies and to know their medical record [2, 22–24, 28–34]. This diagnosis is met when the following four major criteria are met [17, 18]: history of rapid ascent followed by loss of consciousness; air in the left side of the heart and circle of Willis; low probability of post-mortem decompression artefact (PMDA) or decomposition; and mediastinal or subcutaneous emphysema limited to the perithoracic area and/or pneumothorax.

Studies describing some of the tissue lesions characteristic of PBt are scarce and based on the use of classical histological techniques [12, 17, 18, 22–24, 28, 34]. Other studies have used experimental models that induce PBt in animals, commonly rabbits, in hyperbaric chambers [35]. Recently, a histomorphometric analysis of pulmonary tissue has been proposed as a technique for providing objective data on alveolar distension in cases of asphyxia in humans [36, 37]. However, to date, there are no studies using quantitative techniques to investigate the presence of air in the lungs in cases of PBt.

Based on the PBt physiopathology, the aim of this study was demonstrate and quantify the presence of abnormal AS in pulmonary tissues by histomorphometric digital analysis in order to verify whether they can be used for the differential diagnosis in SCUBA diving fatalities.

## Materials and methods

The study was developed in collaboration with the Institute of Legal Medicine and Forensic Sciences of Catalonia (ILMFSC), the Department of Medical Sciences of the University of Girona, and the Special Group for Underwater Activities of the Spanish Civil Guard. It was authorized on September 20, 2018 by the Teaching and Research Commission of ILMFSC and the Research Ethical Committee of Bellvitge University Hospital, Barcelona (Registry number PR328/18).

### Data sources

Our sources of information included data obtained from police technical reports and the forensic pathology service. All victims were diving for recreational purpose and were dependent on gas supply: eleven divers used cylinders containing compressed air as their sole gas supply, and only one diver used an additional supply of Trimix (a mixture of nitrogen, oxygen and helium) in separate cylinders. A detailed summary of the main subject, technical characteristics and sequence of events of all cases is shown in Tables 1 and 2, respectively.

**Table 1:** Summary of data from police technical reports

**Case**	**Age (years)**	**Sex**	**Experience**	**Certification level**	**Breathing apparatus**	**Dive profile (ascent)**	**Depth (m)**	**Loss of consciousness**
1	41	M	Beginner	Basic	Scuba	Rapid	12	S. post-dive
2	46	M	Experienced	Basic	Scuba	Rapid	10	S. post-dive
3	60	M	Experienced	Advanced	Scuba	Rapid	26	S. post-dive
4	57	M	Unknown	Unknown	Scuba	Rapid	12	Out of water
5	61	M	Experienced	Advanced	Scuba	Rapid	22	S. post-dive
6	59	M	Unknown	Unknown	Scuba	Rapid	12	S. post-dive
7	55	M	Experienced	Basic	Scuba	Rapid	45	S. post-dive
8	47	M	Beginner	Basic	Scuba	Controlled	–	Underwater
9	41	M	Experienced	Technical	Rebreather	Controlled	65	Underwater
10	54	M	Beginner	Unknown	Scuba	Controlled	12–15	Underwater
11	62	M	Experienced	Advanced	Scuba	No ascent	9	Cove
12	66	M	Experienced	Advanced	Scuba	Controlled	29	Underwater

*M* male, *F* female, *Beginner*, non or few experienced diver, *Experienced* experienced diver. Depth (in meters): at the moment of the accident, *S. post-dive* in surface after diving, – unknown

**Table 2:** Sequence analysis of the studied diving fatalities

**Case**	**Trigger**	**Disabling agent**	**Disabling injury**	**Cause of death**
1	Panic	Rapid ascent	Lung overexpansion	PBt/AGE
2	Buoyancy problem	Rapid ascent	Lung overexpansion	PBt/AGE
3	Breathlessness	Rapid ascent	Lung overexpansion	PBt/AGE
4	Panic	Rapid ascent	Lung overexpansion	PBt/AGE
5	Chest discomfort	Rapid ascent	Lung overexpansion	PBt/AGE
6	Panic	Rapid ascent	Lung overexpansion	PBt/AGE
7	Breathlessness	Coronary atherosclerosis	Asphyxia	Drowning
8	Unknown	Coronary atherosclerosis	Asphyxia	Drowning
9	Unknown	Oxygen toxicity	Asphyxia	Drowning
10	Low visibility	Panic	Asphyxia	Drowning
11	Confusion	Entrapment	Asphyxia	Drowning
12	Confusion	Entanglement	Asphyxia	Drowning

*PBt/AGE* arterial gas embolism following pulmonary barotrauma

### Lung ample from SCUBA diving fatalities

This study includes twelve cases of diving deaths recorded on the Mediterranean Sea (Girona coast, Northeast of Spain) between January 2009 and January 2020. Post-mortem, the bodies were immediately refrigerated at 5–6 °C. All the lung samples were obtained from autopsies conducted at the Forensic Pathology Service of ILMFSC, with a post-mortem interval (PMI) of less than 30 h, and by forensic pathologists with experience in diving fatalities.

We studied lung parenchyma samples from a histological point of view corresponding to six divers (mean age at death of 54 years, range of 41–61 years) where PBt/AGE was diagnosed as the cause of death according to the protocols and specific recommendations for this type of death [2, 17, 18, 22, 23, 29]. In order to compare these results with the histological pattern that can be found in other types of SCUBA diving-related fatalities and as positive controls, we selected six divers (mean age of death of 54 years, range of 41–66 years) whose death was due to drowning in saltwater and without a diagnosis PBt/AGE. The negative control group included six cases of non-SCUBA divers (mean age at death of 42 years, range of 23–55 years) whose cause of death was intracerebral haemorrhage in which a low probability of lung distension was expected.

Exclusion criteria for this study were included age over 65 years; PMI longer than 30 h; use of high-pressure oxygen in cardiopulmonary resuscitation (CPR) advanced manoeuvers; admission to the intensive care unit of a medical centre; and pneumological diseases showing chronic pulmonary emphysema according to medical records and standard histological results. These exclusion criteria were established in accordance with different studies published describing the presence of air secondary to age (senile lung emphysema), decomposition, CPR advanced manoeuvers and pre-existing pathologies [24, 33, 38].

### Routine histology

During the autopsies, randomly chosen samples from different lobes of the lung in non-hypostatic central and peripheral areas were taken and placed in 10% formalin. The paraffin-embedded lung samples were cut into 5-µm sections and stained with haematoxylin and eosin (HE) for routine histological examination by our Forensic Pathology Service.

### Image analysis

After routine histological analysis, we reassessed these samples by direct optical microscopy Leica DMRXA and selected seventy-two slides. Six microscope fields were randomly selected (10 × magnification) for each slide, and a total of 432 fields are photographed with a Point Grey Flea3 CMOS USB3.0 digital camera with 12MPx coupled with optical microscopy.

**Fig. 1:**
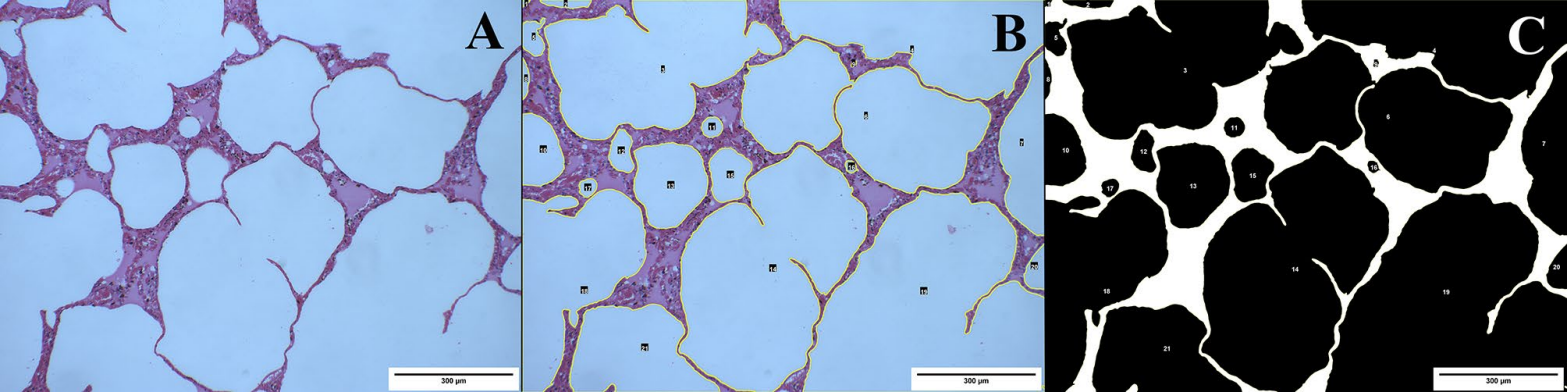
Image corresponding to lung tissue (HE, 10 ×) (**A**) and its digital analysis by the specific Image J Macro used (**B**, **C**). The image shows segmentation process where air spaces (AS) are accurately defined (yellow line) **(B)**. It shows the binarization process of the previous image where AS are identified as black areas (**C**)

**Fig. 2:**
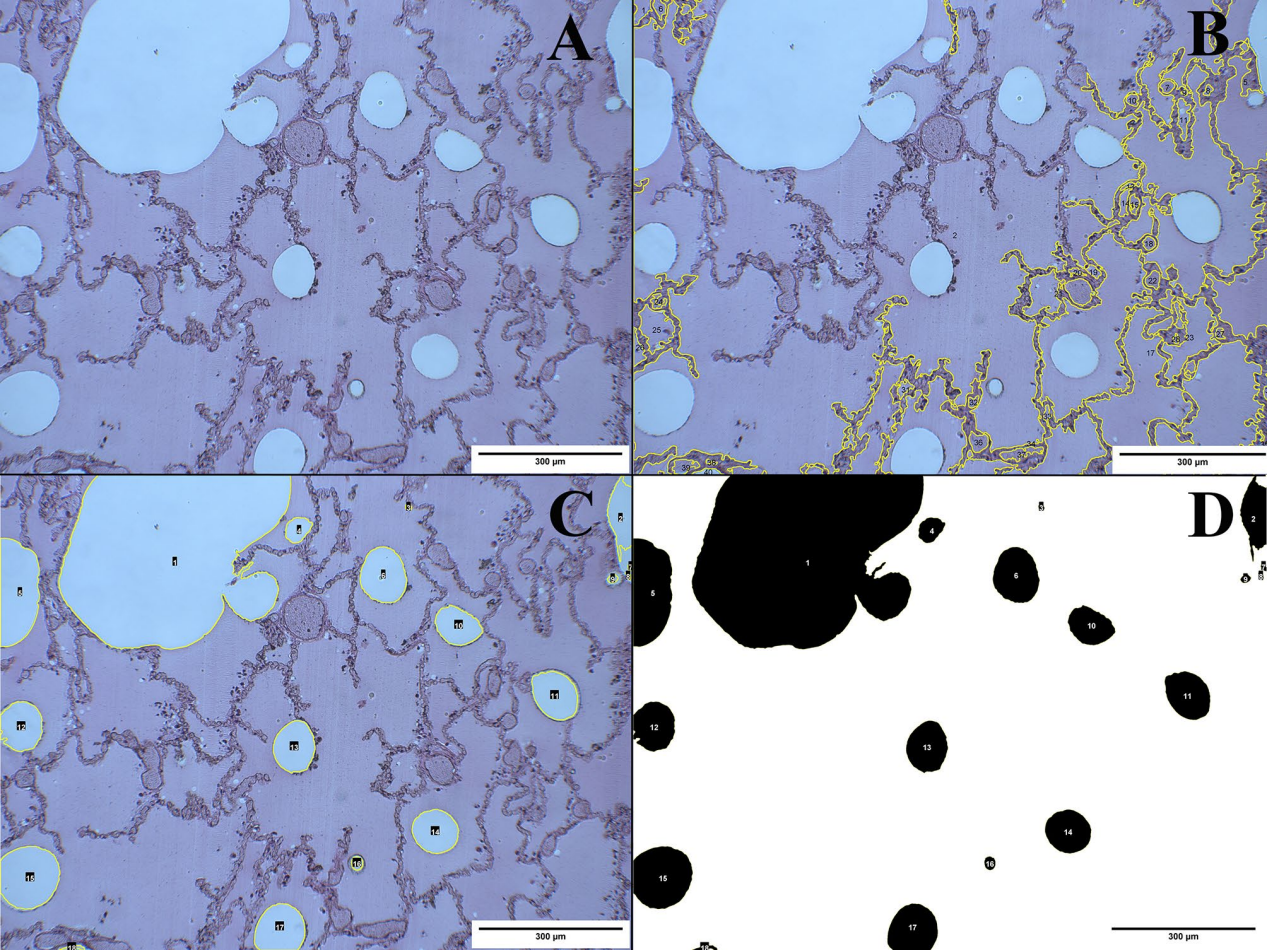
Image corresponding to lung tissue (HE, 10 ×) (**A**) and its digital analysis by the specific Image J Macro used (**B**, **C**, **D**). In this case, we show a wrong segmentation defined by the yellow line (**B**), its manual adjustment in order to well define the air spaces (**C**) and the binarization process of the correct segmentation (**D**)

Digital photographs were analysed using open-source image analysis software (FIJI ImageJ2, National Institutes of Health). This software can easily and carefully detect AS in each field by specific Image J Macro (for segmentation and posterior measurement, an adaptive threshold was applied, based on levels of red, on the parenchymal component of each image, with automatic posterior binarization). We defined an AS as an air collection, located in an intra-alveolar or extra-alveolar space and with a minimum area of 100 µm^2^ (Fig. 1). All AS present in each sample were identified, including those that were incomplete at the edges of the field. Conversely, the presence of oedema, blood vessels or intra-alveolar cells was automatically excluded and not identified, but in some cases, the AS had to be manually selected in order to exclude artefacts (Fig. 2).

The following parameters were measured for each field: count of AS (CAS); total area occupied by AS (AAS); percentage (%) of the field occupied by the AS compared to the rest of the parenchyma (PAS); and maximum diameter (MaxDAS) and minimum diameter (MinDAS) of each air collection. Finally, these parameters were automatically calculated and exported to a MS Excel table.

### Statistical analysis

Normality and equality of variance were observed using the Shapiro–Wilk and Levene tests. ANOVA test and post hoc analysis were performed to compare means between groups. Receiver operating characteristic (ROC) curve analysis was performed to establish a cut-off value to evaluate the sensitivity and specificity of different parameters. SPSS software (v25.0, IBM, Armonk, NY) was used for the statistical analysis.

## Results

A brief summary of histological results from all cases is shown in Table 3.

**Table 3:** Routine histopathological findings

**Case**	**Group**	**Focal emphysema**	**Subpleural bullae**	**Oedema**	**Alveolar haemorrhage**	**Pulmonary congestion**
1	PBt/AGE	✔		✔		✔
2	PBt/AGE		✔	✔	✔	
3	PBt/AGE	✔		✔		✔
4	PBt/AGE	✔	✔	✔		
5	PBt/AGE			✔		✔
6	PBt/AGE			✔		✔
7	Drowning	✔		✔		✔
8	Drowning	✔		✔		✔
9	Drowning			✔		✔
10	Drowning	✔		✔		✔
11	Drowning			✔		✔
12	Drowning			✔		✔
13	CIB			✔		✔
14	CIB			✔		✔
15	CIB			✔		
16	CIB			✔		
17	CIB			✔		✔
18	CIB			✔		✔

*PBt/AGE* arterial gas embolism following pulmonary barotrauma, *CIB* cerebral internal bleeding

### Morphometric analysis

**Fig. 3:**
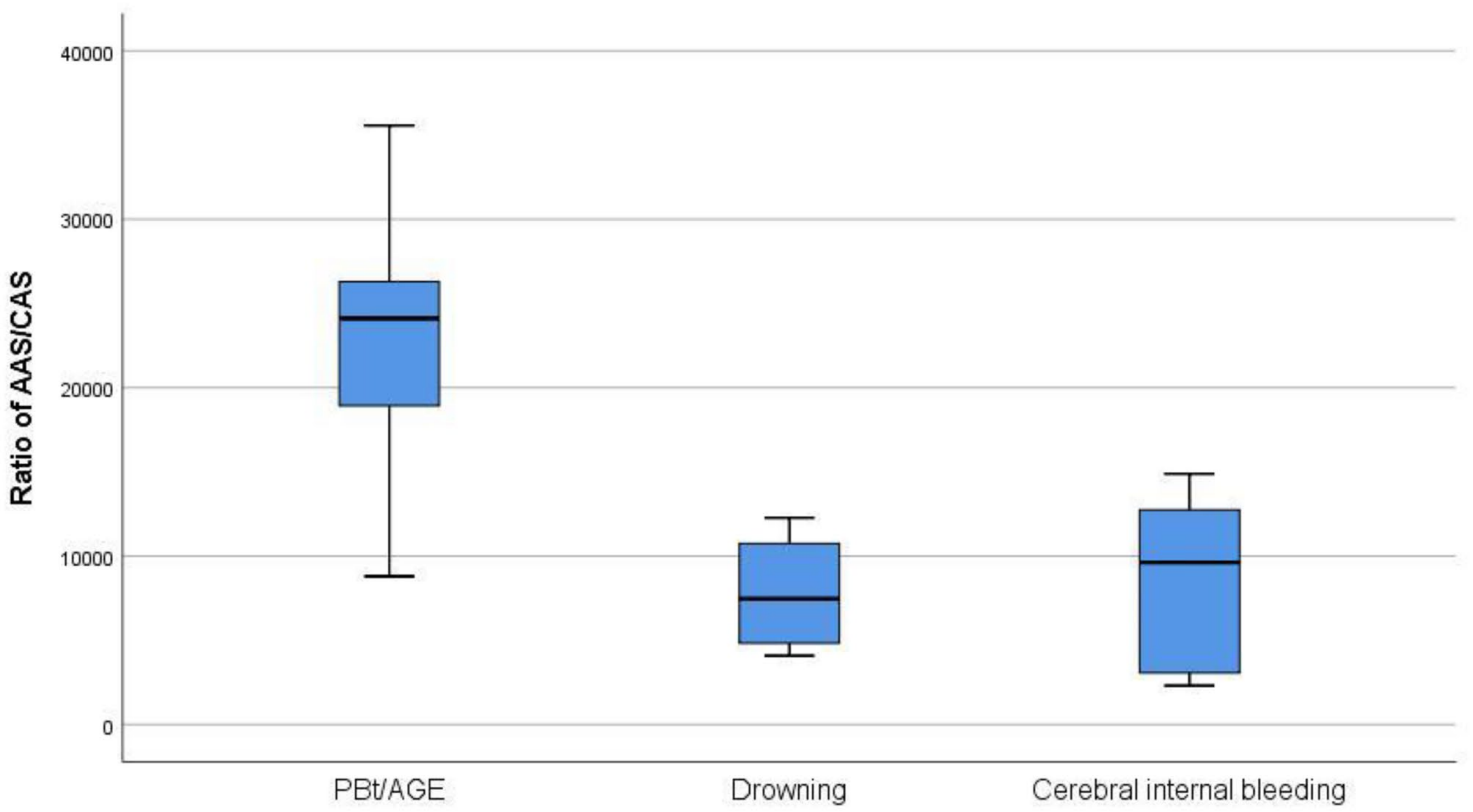
Boxplot of air total spaces area mean ratio and air spaces count (AAS/CAS)

The ANOVA test and post hoc analysis results are summarized in Table 4. No significant differences were observed in AS characteristics between the saltwater drownings group and the intracerebral haemorrhage group. Differences were observed in the mean area of AS (AAS), mean area ratio for count of AS (AAS/CAS) and in percentage of AS (PAS) for each field compared to the rest of the parenchyma (*p* < 0.01) between the PBt/AGE and the drownings groups (Fig. 3). There was a slight difference in AS maximum and minimum diameters (maximum DAS and minimum DAS) (*p* < 0.05) between the PBt/AGE and drownings groups.

**Table 4:** Mean (standard deviation) for count, area, percentage and diameter of air spaces. An ANOVA test and Bonferroni post hoc analysis between groups were performed taking drownings as the reference group. *p < 0.05, **p < 0.01

**Parameter**	**PBt/AGE**	**Drowning**	**CIB**
Mean CAS (units)	1503 (606)	1973 (665)	2312 (1077)
Mean CAS per field (units)	62.60 (25.26)	85.80 (33.04)	96.32 (44.89)
Mean AAS (µm^2^)	856,950 (102,849) **	494,977 (106,953)	547,856 (201,029)
Mean CAS/AAS (µm^2^)	22,968 (8883) **	7807(3536)	8708 (5094)
Mean PAS (%)	65.47 (7.86) **	37.82 (8.17)	41.86 (15.36)
Maximum DAS (µm)	144.54 (32.27) *	92.51 (17.4)	95.90 (35.91)
Minimum DAS (µm)	76.94 (21.35) *	47.33 (12.67)	53.12 (18.84

*PBt/AGE* arterial gas embolism following pulmonary barotrauma, *CIB* cerebral internal bleeding, *AAS* area of air spaces, *CAS* count of air spaces, *PAS* percentage of air spaces, *DAS* diameter of air spaces

**Fig. 4:**
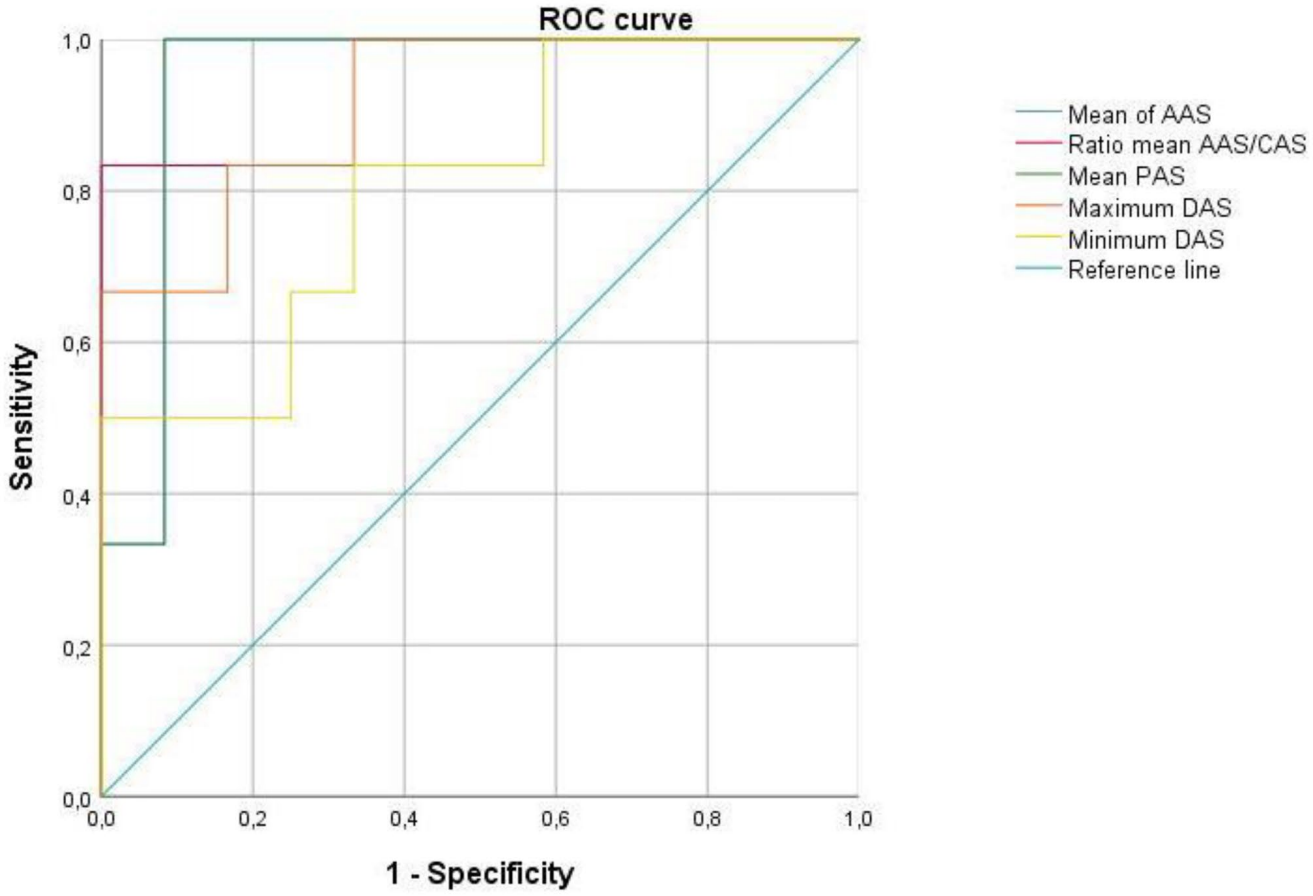
ROC curve analysis of pulmonary barotrauma against other causes of death

ROC curve analysis was performed between PBt/AGE and the other two groups (Table 5 and Fig. 4). The mean AAS and mean PAS were best (AUC of 0.944) for discriminating PBt/AGE from the others with a 100% sensitivity and 91.7% specificity for every cut-off value.

**Table 5:** ROC analysis of different parameters to detect pulmonary barotrauma against other causes of death. Sensitivity and specificity were calculated using different cut-off values for each parameter. *p < 0.05, **p < 0.01

**Parameter**	**Area under**	**Cut-off value**	**Sensitivity**	**Specificity**
Mean AAS	0.944 (**)	718.7 × 10^3^ µm^2^	100%	91.7%
Ratio of mean CAS/AAS	0.903 (**)	16,908	83.3%	100%
Mean PAS	0.944 (**)	54.9%	100%	91.7%
Maximum DAS	0.917 (**)	121.4 µm	83.3%	83.3%
Minimum DAS	0.806 (*)	60.6 µm	83.3%	66.7%

*AAS* area of air spaces, *CAS* count of air spaces, *PAS* percentage of air spaces, *DAS* diameter of air spaces

## Discussion

PBt/AGE is one of the most serious diving accidents that can occur during SCUBA diving ascent if the breath is held and retained inside the lungs while environmental pressure is reduced. Some authorities describe or refer to this as parenchymal haemorrhage, segmental or lobe rupture and even lung bursts [9, 14, 39]. In our series of autopsies, only one case of alveolar haemorrhage was identified. This can possibly be explained by the Macklin effect [40–43] as the breathing gas excess looks for a natural pathway exit while maintaining certain anatomical integrity due to the fact that the air accesses extra-pulmonary areas located in the subcutaneous tissues, pleural cavity, mediastinum, peritoneum and even the retroperitoneum via the perivascular sheaths of the lung.

Our results also show that none of the analysed histological parameters allowed us to distinguish between saltwater drownings and controls. Although some pathological changes have been described in drowning as emphysematous-like alveolar patterns or “emphysema aquosum” [12, 44, 45], other authors believe that there are no specific histological changes [23, 46]. Recent studies suggest that acute pulmonary emphysema is not a typical sign of death by saltwater drowning [37]. Conversely, in the PBt/AGE group, our results show an emphysematous pattern that is different from the other analysed groups and congruent with bronchoalveolar emphysema. Morphologically, we observed that AS occupy a larger area of distribution, even overlapping each other, and with an over-distension that is both intra- and extra-alveolar. These results demonstrate the presence of a significantly larger AS in the pulmonary parenchyma of divers that died within a context of a PBt/AGE. We believe that this might be related to the intrapulmonary high-pressure air retention. These results differ from the histology we found in cases of death by drowning and the control group.

Autopsy evidence from cases of fatal PBt/AGE needs to be interpreted with caution [24] because the macro- or microscopic findings may not be definitively attributed to gas formation as a result of the reduction in environment pressure [46]. Admittedly, the presence of intravascular air is not uncommon in autopsies of SCUBA diving deaths, and it is not specific to PBt/AGE [2, 17, 18]; it can also be due to explosive DCS, post-mortem decompression artefact (PMDA), decomposition and/or resuscitation. It is important to distinguish AGE from DCS, although they can occasionally occur simultaneously [11]. In all SCUBA divers studied herein, we were able to remove DCS as a diagnosis because these divers did not stay under water long enough or go deep enough to develop this dysbaric disorder. Therefore, we believe that PMDA, as documented in the police report, is improbable. In addition, performing autopsies with a PMI of less than 30 h avoided the artefacts of putrefaction or decomposition in our study. Even so, some authorities differentiate between in vivo gas embolism and putrefaction by using gas composition analysis [47], which is a good alternative to avoid the artefact of putrefaction as long as the necessary technical resources are available. Similarly, although resuscitation efforts secondary-to-CPR manoeuvers may rarely simulate larger volumes usually seen with PBt [2, 17, 18], well-controlled animal experiments suggest that in fatal SCUBA diving accidents, subcutaneous emphysema should not be mistaken as diagnostic criteria for barotrauma, because it may be an artefact caused by the resuscitation manoeuvers [30].

Together with a history of rapid ascent as registered in the dive profile, the presence of air in the systemic circulation is one of the key criteria for diagnosing PBt/AGE [17, 18, 29]. Although some studies have described technical modifications that demonstrate this in various organs at autopsy [33], the use of post-mortem imaging techniques should be used when available. Unfortunately, these radiological and macroscopic techniques may not be conclusive to show air existence because most autopsies are performed by pathologists who are not familiar with diving-related injuries. All of this justifies the need to use supplementary analyses to help confirm the radiological and macroscopic findings and differentiate PBt/AGE from other pathologies characteristic of diving, particularly drowning. This is especially important for the medical examiner or coroner’s office, as most diving-related deaths will undergo autopsies at these facilities and not at a large university hospital pathology department.

In addition, a careful histological examination is required for distinguishing these from positive pressure ventilation; pre-existing disease such as chronic pulmonary emphysema; and death due to cardiac oedema, acute asthma or intoxications [48, 49]. In particular, and although in all our cases toxicological analyses were negative, one should remember that inhalational drug abuse (cocaine) and inhalation of certain gases can also cause PBt [50, 51] and, thus, results in a misinterpretation of histopathological findings.

### Conclusions

Our findings suggest a direct association between PBt and over-distension of AS in pulmonary tissues that can be shown with histomorphometric digital analysis. This study found that an increased histopathological pattern of AS, congruent which an bronchoalveolar emphysema, is a possible major diagnostic criterion for PBt/AGE, including a detailed dive profile, and the radiological and/or macroscopic findings described by Lawrence and Cooke [17, 18]. All of this will provide insights for investigation of fatal SCUBA diving incidents.

### Limitations and perspectives

The heterogeneous distribution pattern of AS described in emphysematous lungs makes it difficult to find strong evidence for a pathognomonic diagnosis. Therefore, a multidisciplinary investigation including the deceased person’s dive profile, specific autopsy techniques and/or post-mortem radiographic imaging must be performed. The histological findings described herein should be with future additional cases and one must consider that pulmonary barotrauma in SCUBA diving fatalities is, in general, uncommon.

## Key points

1. Despite drowning being the most common cause of death, a differential diagnosis is mandatory.
2. A qualified investigation of diving fatalities requires a careful histological examination.
3. Alveolar tears provide a pathway for the entrance of air into the stroma resulting in interstitial emphysema.
4. Histomorphometric analysis allows one to diagnose pulmonary barotrauma with over-distension of the air spaces.
